# Integration of a standardized pharmacogenomic platform for clinical decision support at Boston Children's Hospital

**DOI:** 10.1186/1753-6561-6-S6-P5

**Published:** 2012-10-01

**Authors:** Catherine Brownstein, Vincent A Fusaro, Sarah Savage, Catherine Clinton, Kenneth Mandl, David Margulies, Wendy Wolf, Shannon Manzi

**Affiliations:** 1 Boston Children's Hospital, Boston, MA 02115, USA; 2 Center for Biomedical Informatics, Harvard Medical School, Boston, MA 02115, USA

## 

The Clinical Pharmacogenomics Service (CPS) at Boston Children's Hospital (BCH) was established to use genomic information to make pediatric medications safer. Nearly one-quarter of outpatients are prescribed one or more drugs with genetic information in the FDA label [1]. However, there are still important barriers that must be overcome for routine pharmacogenomic (PGx) clinical use: (1) identification of clinically significant variants, (2) knowledge of variant genotype prior to prescribing medication, and (3) integration with current electronic health record (EHR) systems. To tackle these challenges at BCH, the CPS decided to standardize thiopurine *S*-methyltransferase (TPMT) testing hospital-wide.

TPMT is best known for its role in the catalyzing the *S*-methylation of the thiopurine drugs such as azathioprine, 6-mercaptopurine and 6-thioguanine. Approximately 13% of Caucasians and African Americans are heterozygous and have reduced TPMT activity, while approximately 0.3% are completely deficient. Defects in the *TPMT* gene can lead to decreased methylation and excessive levels of the toxic thioguanine nucleotides, particularly with azathioprine and 6-mercaptopurine, and are at risk for bone marrow suppression.

Although the FDA drug label recommends testing for TPMT deficiency prior to dosing and the PharmGKB CPIC group published a guideline [2] with a recommended dosing strategy and interpretation, testing is not universal because these guidelines are difficult to translate into a clinical decision support (CDS) system and integrate with the EHRs. We developed models and specifications to execute PGx CDS rules based on a patient's genotype. Rules are modeled at four levels of abstraction: (1) unstructured (narrative), (2) semi-structured, (3) structured, and (4) executable.

As genomic sequencing becomes routine, standardized methods to interpret the data and make clinical decisions are paramount. In conjunction with the BCH DNA Diagnostic Laboratory, we streamlined the TPMT testing process to fit into the usual clinical routine (including ordering, testing in-house and return of results to the clinician). We consolidated all genetic sequencing testing into a single clinical workflow (blood to report) that is run, analyzed and interpreted in a Clinical Laboratory Improvement Amendments (CLIA) certified laboratory using the codified CDS rules. The interpretation reports are generated automatically directly from the genotype calls and then manually reviewed for accuracy. Once cleared by the laboratory director, the reports are uploaded into the EHR (Cerner). Specialty flow sheets enable providers to easily view the allele status and interpretation report. We intend to expand the PGx platform to include additional drug/gene pairs.
